# Flexural Behavior Characteristics of Steel Tubes Filled with SFRCCs Incorporating Recycled Materials

**DOI:** 10.3390/ma16051958

**Published:** 2023-02-27

**Authors:** Hyun-Do Yun, Wan-Shin Park, Young-Il Jang, Sun-Woo Kim

**Affiliations:** 1 Department of Architectural Engineering, Chungnam National University, Daejeon 34134, Republic of Korea; 2 Department of Construction Engineering Education, Chungnam National University, Daejeon 34134, Republic of Korea

**Keywords:** SFRCCs, steel tube, flexural behavior, indentation, deformation capacity

## Abstract

This study deals with the effect of fly ash and recycled sand on the flexural behavior of SFRCCs (steel fiber-reinforced cementitious composites)-filled steel tubes. As a result of the compressive test, the elastic modulus was reduced by the addition of micro steel fiber, and the fly ash and recycled sand replacement decreased the elastic modulus and increased the Poisson’s ratio. As a result of the bending and direct tensile tests, strength enhancement by the incorporation of micro steel fibers was observed, and a smooth descending curve was confirmed after initial cracking. As a result of the flexural test on the FRCC-filled steel tube, the peak load of all specimens was similar, and the applicability of the equation presented by AISC was high. The deformation capacity of the steel tube filled with SFRCCs was slightly improved. As the elastic modulus of the FRCC material lowered and the Poisson’s ratio increased, the denting depth of the test specimen deepened. This is believed to be due to the large deformation of the cementitious composite material under local pressure due to the low elastic modulus. From the results of the deformation capacities of the FRCC-filled steel tubes, it was confirmed that the contribution of indentation to the energy dissipation capacity of steel tubes filled with SFRCCs was high. From the comparison of the strain values of the steel tubes, in the steel tube filled with SFRCC incorporating recycled materials, the damage was properly distributed between the loading point and both ends through crack dispersion, and consequently, rapid curvature changes did not occur at both ends.

## 1. Introduction

Concrete-filled steel tube (CFST) members have recently been widely used for structural applications, as they have superior performance compared to conventional reinforced concrete (RC) and steel tube members in terms of not only mechanical properties, such as high strength and stiffness [1,2,3,4,5,6,7,8,9,10], but also their excellent ductility, energy dissipation ability [11,12,13,14,15] and fire resistance [16,17,18,19,20]. The outstanding performance of CFSTs is due to the complex action between concrete and steel, and the strength of the infilled concrete is greatly improved due to the confining effect of the outer steel tube.

Recently, studies on the structural performance of CFST members using steel-fiber-reinforced cement composites have been conducted in various fields. In fiber-reinforced cementitious composites, the reinforcing fiber exists in the form of short discrete fibers in the cement matrix and enhances the dispersion of microcracks and tensile strength, depending on the tensile strength of the fiber and its adhesion behavior with the cement matrix [21]. The reinforcing fibers can improve brittle fracture characteristics such as the low tensile strength and deformability of cement composites. In addition, engineered cementitious composite (ECC) [22], high-performance fiber-reinforced cement-based composite (HPFRCC) [23] and strain-hardening cement-based composite (SHCC) [24] show strain-hardening characteristics after initial cracking. Among the reinforcing fibers, steel fibers can improve brittle fracture characteristics such as the tensile strength and deformation capacity of concrete more distinctly due to their large cross-sectional area and high strength and stiffness compared to micro-synthetic fibers, such as polyethylene (PE) or polyvinyl alcohol (PVA). Steel fibers show different behavior characteristics depending on the aspect ratio or shape of the fiber [25,26]. In addition to the material properties of fiber-reinforced cementitious composites, many studies on the structural behavior characteristics of steel-fiber-reinforced concrete-filled tubes have also been conducted [27,28,29,30,31,32]. Moreover, studies are being conducted to improve material and structural behavior characteristics by utilizing recycled resources, such as waste lathe fibers or steel wires from waste tires as reinforcing fibers in concrete [33,34,35,36].

Regarding the recycling of waste, studies have been conducted to replace the main constituent materials of concrete, such as aggregate and cement, with recycled materials. In the case of aggregates that are consumed in large amounts in the production of concrete, not only recycled concrete aggregate [37,38,39,40,41,42] obtained by crushing waste concrete but also various industrial and household wastes, such as ground glass powder, crushed waste glass [43,44,45] and marble wastes, are the subjects of research on the utilization of alternative materials [46]. As part of an effort to reduce CO_2_ emissions from the cement manufacturing process, industrial by-products such as fly ash and blast furnace slag, as well as household waste such as waste glass or waste marble powder, have been used as cement substitutes for cement-based materials, and the structural application of the materials have been evaluated [47,48,49,50,51]. It has also been reported that the incorporation of fly ash into FRCC enhances the adhesion between the reinforcing fibers and the cement matrix, thereby improving the tensile performance of fiber-reinforced cement composites [52,53].

The CFST member can partially restrain local buckling occurring in steel tubes and thus is effective in delaying or preventing severe structural damage and collapse due to local forces, such as collisions with vehicles or ships [54]. When a hollow steel tube member is subjected to a local impact load, the deformation by the impact energy is distributed to indentation and overall bending deformation [55], resulting in a high energy dissipation capacity [56,57,58]. However, in the case of the CFST member, indentation hardly occurs due to filled concrete, resulting in the formation of a local plastic hinge at an early stage, significantly reducing the energy dissipation ability of the member [59,60].

As described above, because indentation is dominant in hollow steel tubes, a hammer with a knife-edge shape is commonly used in the experiment [56,57,58]. On the other hand, because indentation rarely occurs in CFST, the influences of the different shapes of drop hammers on the behavior of CFSTs may not be significant [61], and various shapes of drop hammers are used in the experiment [59,60,62]. However, FRC and FRCC mixtures show a decrease in the elastic modulus as the fiber volume fraction increases, and in the case of FRCC, the decrease in the elastic modulus is greater than that of FRC because there is no coarse aggregate [63]. Such a decrease in elastic modulus can indicate an increase in the deformation of FRCC, so when an experiment is performed using a drop hammer with a knife-edge shape, the indentation depth of the FRCC-filled tube can increase. However, in studies on FRC-filled steel tubes [64,65], this indentation effect has not been considered. Moreover, only the overall bending performance has been evaluated, and the deformation capacity affected by indentation has not been considered.

In this study, Steel-Fiber-Reinforced Cement Composites (SFRCCs), which improve the brittle fracture characteristics, crack distribution and fracture toughness of existing cement composites such as concrete, were applied as steel tube fillers, and the flexural performance was evaluated experimentally. In the case of ECC or HPFRCC, fine silica sand is generally used to maximize the adhesion between the reinforcing fibers and the matrix, and the cement composite consequently exhibits strain hardening characteristics after cracking. However, considering that the material in this study was applied as a steel tube filler, the application of silica sand lowers the economic feasibility. Therefore, in this study, a fine aggregate was applied in consideration of the performance and economic feasibility when manufacturing fillers for steel tubes. As part of efforts to expand the application of recycled aggregate as construction waste increases due to redevelopment projects, some of the natural fine aggregate was replaced with recycled fine aggregate when manufacturing FRCCs. To evaluate the indentation effect during the lateral loading of FRCC-filled steel tubes, a hammer with a knife-edge shape was used for the flexural test. The contributions of bending and indentation on the deformation capacities of FRCC-filled steel tubes were compared and discussed.

## 2. Experimental Program

### 2.1. Test Specimens

**Figure 1:**
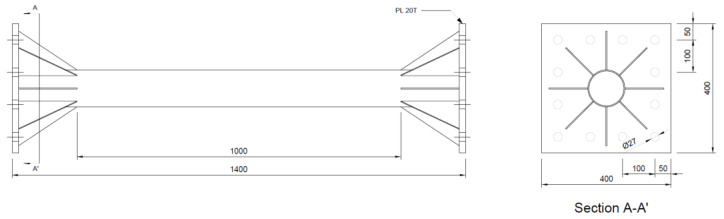
Details of steel tube specimen (Unit: mm).

In this study, to evaluate the effect of fly ash and recycled fine aggregate on the flexural behavior of a steel tube filled with SFRCCs, a total of three specimens were planned with the type of steel tube filler as a variable. The steel tube was S355 grade [66] with a yield strength of 415 MPa, a tensile strength of 505 MPa and an elongation of 30% (test value provided by the manufacturer). The shape and dimensions of the steel tube are shown in Figure 1, and the conditions and variables of the specimen are summarized in Table 1.

### 2.2. Materials

In this study, through a preliminary experiment on the mechanical properties of SFRCCs according to the replacement ratio of recycled materials, the volume fraction of steel fiber was set to 1.0% of the cement volume ratio to ensure optimal mixing and tensile performance. In addition, in a preliminary experiment, a 25% fly ash replacement rate and a 50% recycled fine aggregate replacement rate, which are levels that do not decrease the mechanical performance (compression, bending and tension) of cement composites by more than 10%, were applied. The water-to-binder ratio was set to 0.4. Through numerous preliminary experiments on the mixing performance, flowability and mechanical properties of steel-fiber-reinforced cementitious composites, the best design mixture was derived, which is shown in Table 2. As presented in Table 2, a total of three mixtures were considered: an OPC mixture using ordinary Portland cement, an OPCF mixture mixed with steel fiber and an F25R50F mixture in which the cement and natural aggregate of the OPCF mixture are substituted with 25% fly ash and 50% recycled fine aggregate, respectively.

**Figure 2:**
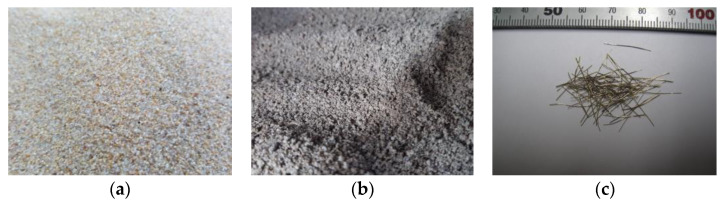
Fine aggregates and steel fiber used in this study: (**a**) Natural sand; (**b**) Recycled sand; (**c**) Steel fiber.

**Figure 3:**
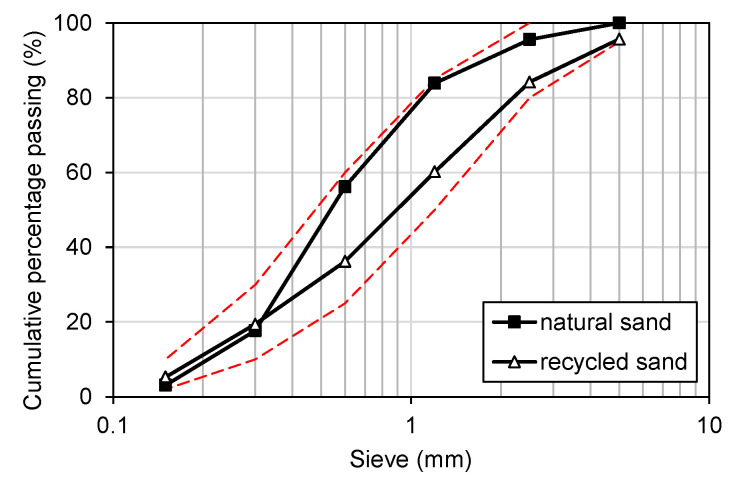
Sieve analysis result.

As cementitious materials, Class 1 ordinary Portland cement that satisfies KS L 5201 [67] and Class F fly ash produced in Boryeong thermal power plant that satisfies KS L 5405 [68] were used in this study. The natural sand was sea sand, obtained from Jumunjin, Gangwon Province. Recycled sand is an aggregate that is produced by crushing waste concrete during the dismantling process of the existing old RC structure and satisfies KS F 2573 [69]. The fine aggregates and steel fiber used in this study are shown in Figure 2. The percentages of the grain sizes of fine aggregates used in this study were determined by conducting sieve tests as per Korean standard KS F 2502 [70]. Figure 3 shows the size distribution of the fine aggregates. Both natural and recycled sand fall between the upper and lower limit. The chemical composition of fly ash used in this study and the material properties of natural sand and recycled sand are listed in Table 3 and Table 4, respectively, and the physical properties of steel fiber are provided in Table 5.

**Figure 4:**
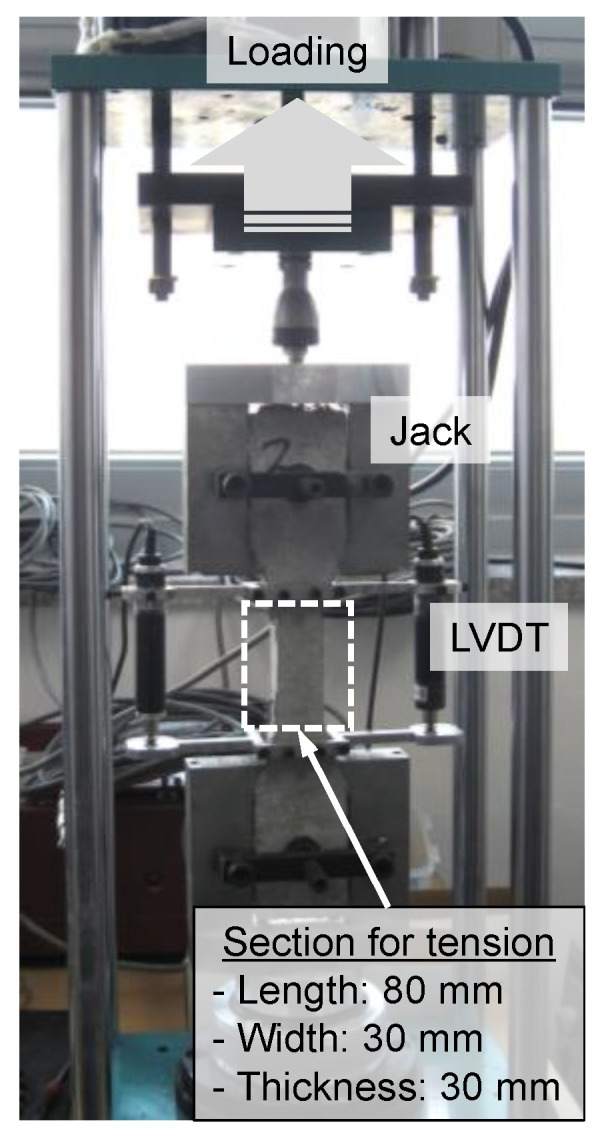
Tensile test set-up.

In this study, to evaluate the mechanical properties of each cement composite, test specimens for compressive and flexural strength tests were manufactured in accordance with KS L ISO 679 [71]. In addition, the direct tensile strength test specimen had a dumbbell shape. The tensile behavior characteristics were quantitatively evaluated using the direct tensile strength test machine shown in Figure 4. All specimens for the evaluation of mechanical properties were demolded after one day of casting and were cured in water in a constant-temperature water bath (20 ± 2 °C) for 28 days.

### 2.3. Mechanical Properties of Cement Composites

In this study, to evaluate the effect of fly ash and recycled aggregate on the bending behavior of SFRCC-filled steel tubes, three types of cement composites for steel tube filling were set as variables, as shown in Table 1 and Table 2. Table 6 lists the mechanical properties of the cement composites for each mixture. The compressive strength showed slight differences of around 50 MPa in all combinations. In the case of the F25R50F combination, which showed the lowest compressive strength, it was about 3.5% lower than that of OPCF, and there was almost no decrease in compressive strength due to the replacement of recycled materials. According to the research results on the effect of fiber mixing on the compressive strength of existing FRCCs, it has been reported that the fiber mixture shows different results depending on the fibers and blends that are used, such as increasing [72,73,74] or decreasing [75,76] the compressive strength due to fiber mixing. In this study, the compressive strength and modulus of elasticity of cement composites tended to decrease somewhat due to the incorporation of steel fibers, and the Poisson’s ratio tended to increase slightly.

**Figure 5:**
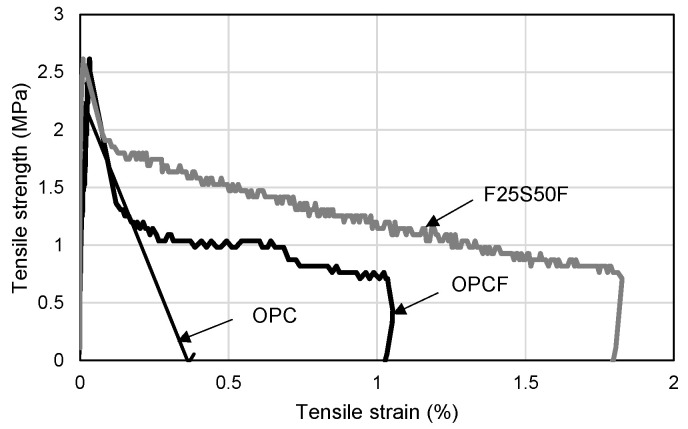
Tensile strength–strain curves of test specimens.

When fine aggregate is used instead of silica sand, it has been reported that it is difficult to express strain hardening characteristics, such as in the case of existing ECC or HPFRCC [77,78,79]. As shown in Figure 5, strain hardening characteristics did not appear in the tensile strength test results of this study. As a result of analyzing the difference in tensile behavior characteristics in each mixture, a brittle fracture occurred in the OPC mixture after the initial tensile crack, whereas the brittle fracture was delayed in the OPCF and F25S50F mixture with steel fibers, due to fiber-bridging action. After the initial tensile crack, the F25S50F mixture showed higher tensile stress than that of the OPCF mixture. This is thought to be due not only to the application of fly ash, which has a higher fineness than cement, but also to the increase in the amount of fine powder in the aggregate generated during the crushing of waste concrete in the process of producing recycled fine aggregate. It is believed that this is because the fly ash and the fine powder enhanced the adhesion area with the cement matrix, which is essential for the adhesion behavior of steel fibers.

In terms of flexural strength, the mixture containing steel fibers had 15 to 16% higher strength than that of the OPC mixture, but the tendency seen in the direct tensile strength test results did not appear. It is believed that this was because the position of the flexural crack that is initially generated and propagated is different for each specimen because no notch is provided in advance, and thus, the bending moment used for calculating the flexural strength shows some deviation.

### 2.4. Flexural Test Method

**Figure 6:**
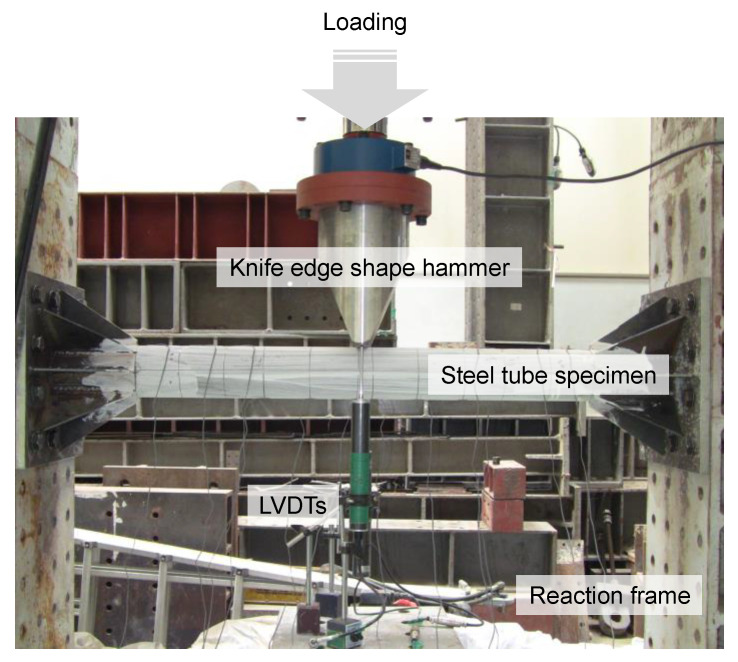
Flexural test set-up.

In this study, the experimental equipment shown in Figure 6 was installed to experimentally evaluate the flexural behavior of SFRCC-filled steel tube members. The loading frame was fixed to the reaction floor, and both ends of the specimen were fastened to the loading frame with high-strength bolts to make the boundary condition a fixed end. Static loading was applied in the vertical downward direction using an oil jack with a capacity of 1000 kN, and monotonic loading was performed via displacement control. To simulate local forces, such as collisions with vehicles or ships, a hammer in the shape of a knife-edge with a width of 30 mm in contact with the specimen was installed at the loading part of the oil jack. To measure the deflection of the SFRCC-filled steel tube specimen, a displacement transducer (LVDT1) was installed at the bottom of the center of the specimen, and a displacement transducer (LVDT2) was additionally installed at the bottom of the knife-edge shape hammer. The displacement difference between displacement transducers (LVDT2-LVDT1) was used to calculate the depth of denting at the loading part of the specimen. In addition, strain gauges were attached to the surface of the steel tube specimen along the longitudinal direction in order to check the yield and plastic deformation characteristics of the steel material at each position during the experiment.

## 3. Experimental Results and Discussion

### 3.1. Failure Modes

**Figure 7:**
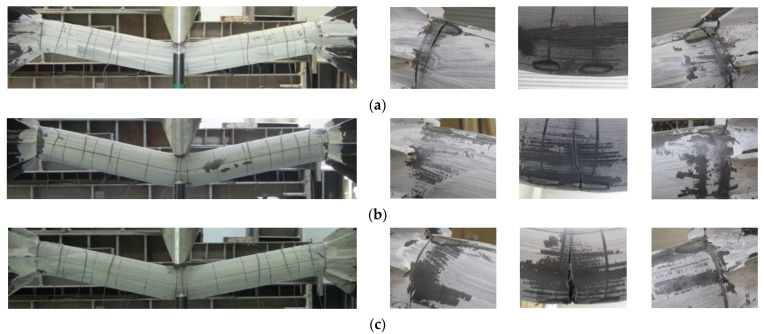
Final failure modes of test specimens: (**a**) STC; (**b**) STF; (**c**) STFR.

**Figure 8:**
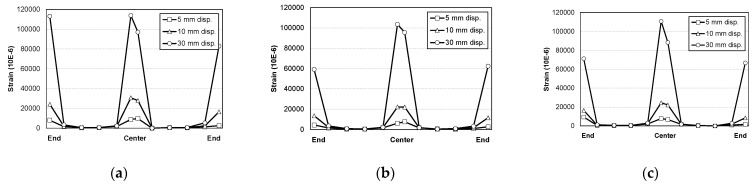
Strain distribution of steel tube: (**a**) STC; (**b**) STF; (**c**) STFR.

Figure 7 shows the final failure modes of the specimens. As shown in Figure 7a, in the STC test specimen, which was a mortar-filled steel tube without mixing steel fiber, fly ash and recycled fine aggregate, local damage at the loading part initially occurred slightly. The damage was then transferred to both ends, resulting in the fracture of the steel at both ends, showing signs of failure. In the STF test specimen filled with SFRCCs (Figure 7b) and the STFR test specimen, which was a steel tube filled with a SFRCC incorporating 25% fly ash and 50% recycled fine aggregate (Figure 7c), fractures occurred at both ends after local damage to the load section. Instead, the bottom of the center of the specimen was fractured and finally failed. In relation to this failure mode, Figure 8 shows the strain distribution of the steel tube specimen according to the increase in displacement. As shown in Figure 8, the steel strain at the center and both ends of the STC specimen was higher than that of the STF and STFR specimens, which were SFRCC-filled steel tubes, at the same displacement. In addition, in the STF and STFR specimens, the strain at both ends was smaller than that in the central part of the steel tube, but the strain at the ends of the STC specimen was similar to that in the central part. It is believed that this was because the SFRCCs were filled in the steel tube, and damage was properly distributed between the central load point and both ends through crack dispersion. Consequently, rapid curvature changes did not occur at both ends, which delayed steel fracturing.

### 3.2. Load–Displacement Relationships

**Figure 9:**
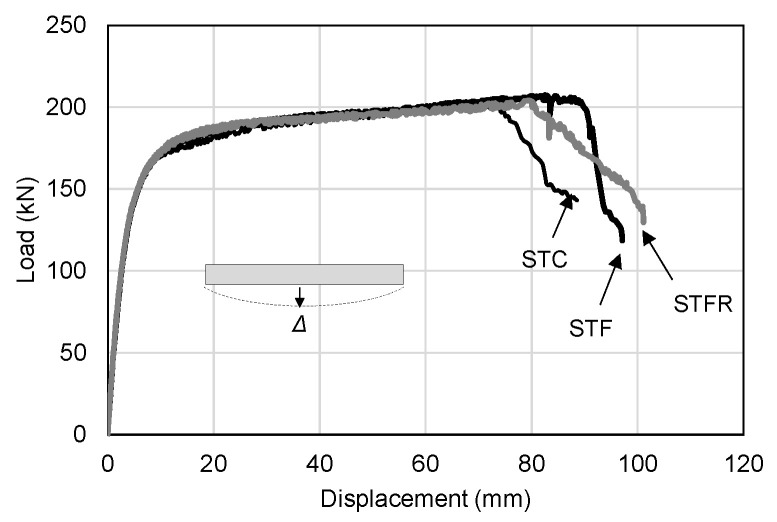
Load vs. deflection curves.

**Figure 10:**
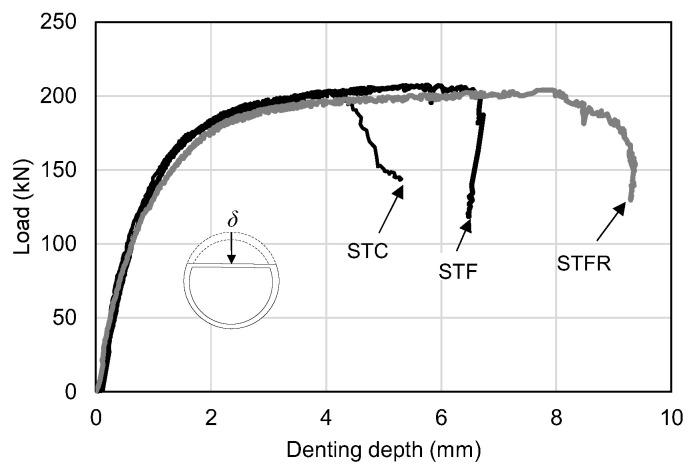
Load vs. denting depth curves.

Figure 9 and Figure 10 show the deflection of the central part and the depth of damage to the load part according to the applied load of the general mortar and SFRCC-filled steel tubes, respectively, and they are summarized and shown in Table 7. The STC test specimen, which was a mortar-filled steel tube without mixing steel fiber, fly ash and recycled fine aggregate, showed a maximum load of 202.42 kN. The local damage depth of the loading part showed a maximum value of 4.05 mm, indicating that the damage at the loading part was insignificant. The STF specimen filled with SFRCCs showed a maximum load of 207.33 kN, and the local damage depth of the loading part at the maximum load was 6.73 mm, which was about 60% deeper than that of the STC specimen. The STFR test specimen, a steel tube filled with a SFRCC with 25% fly ash and 50% recycled aggregate, showed a maximum load of 203.98 kN. The local damage depth of the loading part was up to 9.38 mm, and the damage depth at the loading part greatly increased compared to the STC and STF specimens. This was because, as shown in Table 6, which presents the material test results, the F25S50F mixture showed a lower modulus of elasticity than the OPC and OPCF mixtures. This increased the depth of the primary force part damage and led to a relatively high Poisson’s ratio. As a result, it is judged that the deformation of the steel material due to the lateral expansion was caused secondarily. In addition, it is determined that the damage was redistributed such that the damage was not concentrated on the supporting parts of the member by increasing the damage depth at the applied portion.

In this study, the flexural strength was calculated by applying the plastic stress distribution method of the AISC standard [80], as shown in Equation (1) below.

(1) $$M_{B}=F_{y}Z_{sB}+\frac{0.95f_{c}^{'}Z_{cB}}{2}$$

where $F_{y}$ is the yield strength of the steel tube, $Z_{sB}$ is the plastic section modulus of the steel tube, $f_{c}^{'}$ is the compressive strength of concrete, and $Z_{cB}$ is the plastic section modulus of concrete.

**Figure 11:**
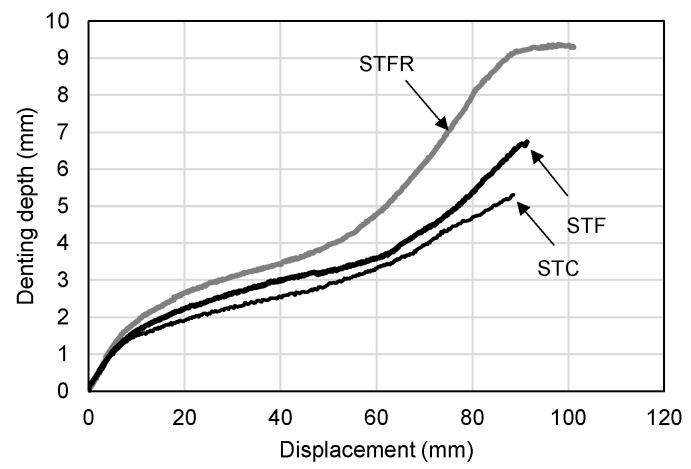
Denting depth vs. deflection curves.

As shown in Table 7, as a result of comparing the experimental values for the cement-composite-filled steel tube with the calculated values according to the AISC standard formula, the error range was within 3%, indicating that the experimental values were properly predicted. Figure 11 shows the relationship between the midspan deflection and denting depth of the SFRCC-filled steel tube. As shown in the figure, it was found that the damage depth increased slightly with the progress of deflection in the STF specimen compared to the STC specimen.

**Figure 12:**
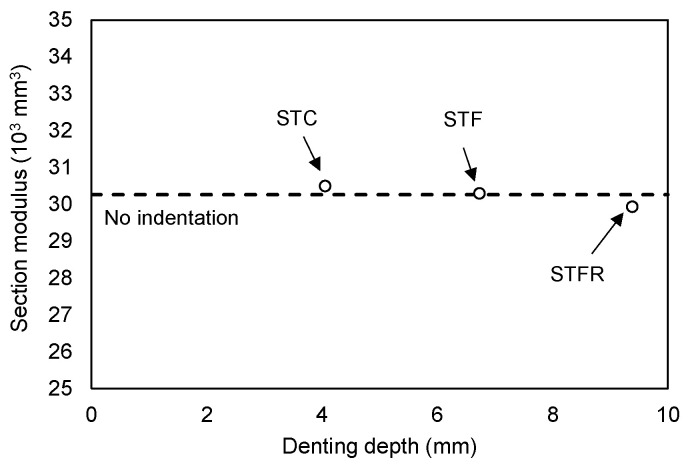
Comparison of section modulus of test specimens.

On the other hand, in the STFR test specimen substituted with fly ash and recycled fine aggregate, which were resource-recycled materials, it was found that the increase in the damage depth at the loading part for deflection increased noticeably. This appears to be because, as described above, the F25S50F mixture showed a lower elastic modulus and a higher Poisson’s ratio than those of the OPC and OPCF mixtures. On the other hand, the results of calculating the moment of inertia of the section of the SFRCC-filled steel tubes showed that it decreased as the denting depth increased, showing a maximum decrease of 9.2% in the case of the STFR test specimen. However, the section modulus applied when calculating the flexural strength showed a difference of 1.0% from the section without damage, as presented in Figure 12. Therefore, it is deemed appropriate to apply the section modulus in the state where no damage occurs when calculating the flexural strength of the CFT, as found in the plastic stress distribution method of the current AISC standard.

### 3.3. Energy Dissipation Characteristics

**Figure 13:**
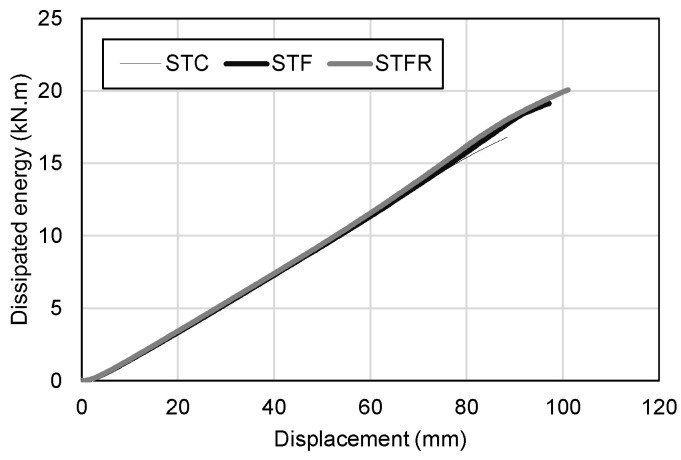
Comparison of the dissipated energy.

Figure 13 shows the total energy dissipation capacity, which is the sum of the energy dissipation capacity due to the bending of the specimen and damage at the loading part. The energy dissipation capacity due to the bending of the specimen and the damage at the loading part was calculated by using the area surrounded by the X-axis and the curve corresponding to each specimen in the load–deflection relationship curve in Figure 9 and the load–damage depth relationship curve in Figure 10, respectively. As shown in Table 7, the STFR test specimen, which was a steel tube filled with an SFRCC replaced with 25% fly ash and 50% recycled fine aggregate, showed the highest energy dissipation capacity at 20.072 kN·m. However, as shown in Figure 13, the tendency to increase the energy dissipation capacity according to deflection was similar regardless of the mixture type.

**Figure 14:**
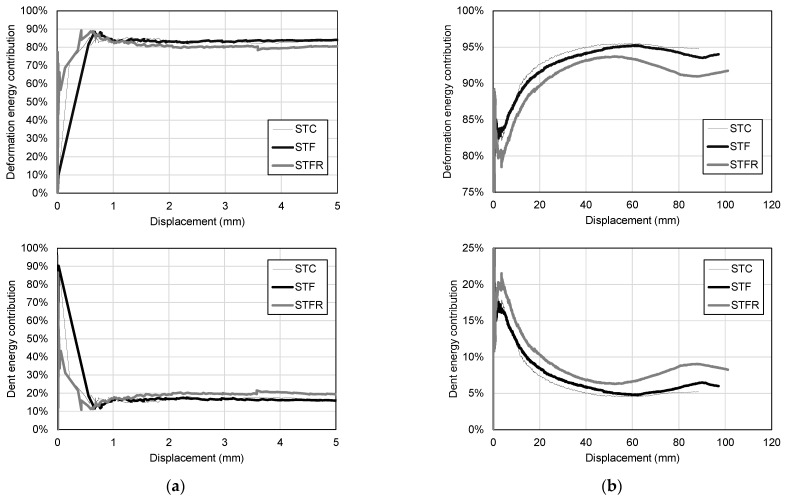
Contribution of deformation and dent to the total energy dissipation capacity: (**a**) Initial stage; (**b**) Overall.

Figure 14 shows the total energy dissipation capacity classified according to the contribution of the bending deformation due to the deflection and denting at the loading part.

As shown in Figure 14a, in all specimens, the energy dissipation capacity at the beginning of the flexural behavior was mostly caused by denting at the loading part, but after the initial crack, the contribution of the energy dissipation capacity by flexural deformation increased rapidly. As deflection increased, as shown in Figure 14b, the contribution of the energy dissipation capacity by flexural deformation increased to more than 90%. More than 80% of the energy dissipation capacity of the SFRCC-filled steel tube was influenced by the midspan deflection, and the contribution by the damage at the loading part was found to be less than 20%. In particular, the STC test specimen, which was a mortar-filled steel tube without steel fiber and recycled materials, showed the lowest contribution to energy dissipation capacity due to damage at the loading part. In the case of the STFR specimen, the contribution to the energy dissipation ability due to damage to the loading part was 9%, which was higher than those of the STC and STF test specimens, and the ability to redistribute local damage was improved.

### 3.4. Steel Strain Characteristics

**Figure 15:**
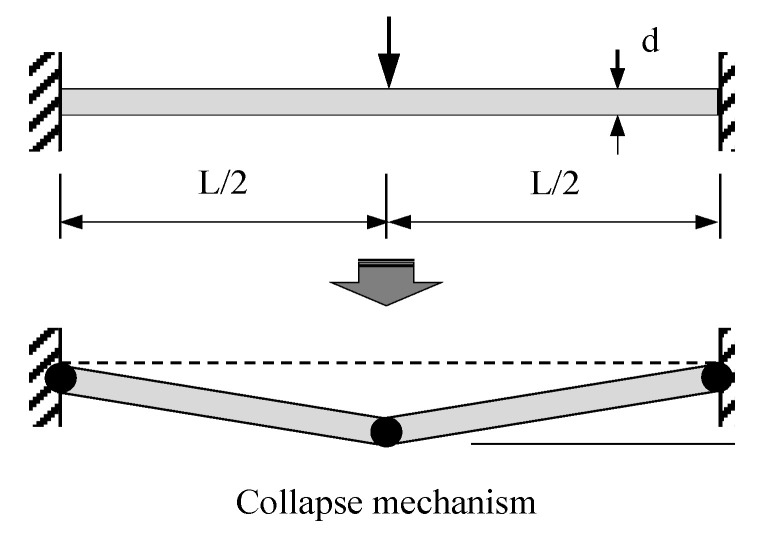
Theoretical plastic collapse mechanism for a clamped beam subjected to transverse load at the mid-span. Reprinted from [81], Copyright 2008, with permission from Elsevier.

In general, in a CFST member, there is almost no local damage to the load part due to the infilled concrete, and it shows failure by forming a plastic hinge due to bending, as shown in Figure 15 [81]. Therefore, in this study, strain gauges were installed at the lower end of the midspan and the upper end of the supporting part of the steel tube to evaluate the strain at the plastic hinge generating area, and the strain value was measured.

**Figure 16:**
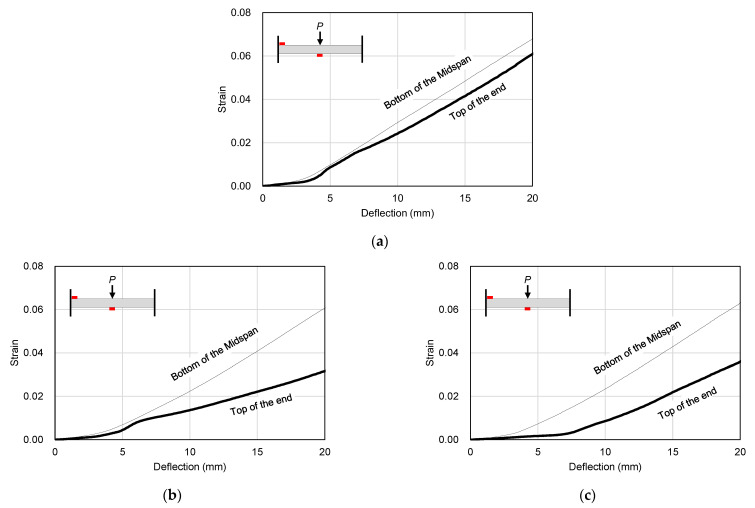
Comparison of strain characteristics of the steel tube: (**a**) STC; (**b**) STF; (**c**) STFR.

Figure 16 shows a comparison of steel strains at the bottom of the midspan and the top of the support part of the specimen. As shown in Figure 16, in all specimens, the steel strain at the top of the supporting part was larger than that at the bottom of the midspan. However, as shown in Figure 16b, after deflection of about 6 mm, the steel strain at the top of the supporting part in the STF specimen mixed with steel fiber was lower than that of the STC specimen. It is believed that this was due to crack dispersion at the loading part due to the incorporation of steel fibers. In the STFR test specimen, which was an SFRCC-filled steel tube with fly ash and recycled fine aggregate, the most stable stress distribution phenomenon was observed; for example, the steel strain at the top of the supporting part started to increase after a deflection of about 6 mm.

## 4. Conclusions

In this study, flexural tests were performed on FRCC-filled steel tubes subjected to one-point loading under the fixed end condition. The effects of using micro steel fiber and recycled materials were experimentally evaluated for the mechanical properties of FRCC materials and the flexural behavior of structural members, and the contribution of bending and indentation to the deformation capacity of FRCC-filled steel tubes was investigated. The conclusions based on the experimental results are summarized as follows.

1. The compressive strength and modulus of elasticity of cement composites tended to decrease, and the Poisson’s ratio tended to increase slightly, due to the incorporation of steel fibers and the substitution of recycled materials. In addition, when steel fibers were mixed, the flexural strength was 15 to 16% higher than that of the OPC mixture, and the retardation of brittle fractures due to the fiber crosslinking stress after the initial tensile crack was exhibited during the tensile test.
2. The STF specimens filled with SFRCCs showed a slight increase in denting depth according to the increase in deflection compared to the STC specimens that were not reinforced with steel fibers. In particular, for the STFR test specimen with the recycled material, the denting depth at the loading part tended to increase noticeably due to the lower elastic modulus and higher Poisson’s ratio of the cementitious composite compared to the OPC mixture.
3. In all specimens, the tendency to increase the energy dissipation capacity according to deflection was similar regardless of the type of mixture for the cement composite. However, in the case of the STFR specimen, the contribution to the energy dissipation capacity due to the damage at the loading part was larger than those of the STC and STF specimens, indicating that the ability to redistribute local damage was improved.
4. In all specimens, the steel strain at the top of the supporting part was larger than that at the bottom of the midspan of the test specimen, which is considered to be due to the dispersion of cracks at the loading part due to the mixing of steel fibers. In particular, in the STFR test specimen, which was an SFRCC-filled steel tube with recycled materials, the lowest steel strain was observed at the top of the supporting part, and steel deformation was delayed the longest. Thus, it is determined that the stress distribution was stable.
5. In this study, based on numerous experiments on the mechanical properties of FRCC, three combinations with the best tensile performance were derived. However, when retrofitting is required at a construction site, materials such as self-compacting cementitious composites with excellent flowability are required. Therefore, verification of the structural applications as well as investigation of SFRCC materials that satisfy both high flowability and mechanical properties are necessary tasks for future research.

## Figures and Tables

**Table 1:** List of test specimens.

Specimen	Steel Tube					Mixture
	L ^1^ (mm)	D ^2^ (mm)	t ^3^ (mm)	L/D	D/t	
STC STF STFR	1000	114.3	3.2	8.75	25.4	OPC OPCF F25S50F

^1^ Net length, ^2^ Diameter, ^3^ Thickness.

**Table 2:** Mix proportions of cement composites.

Mix	W/B (%)	Weight (kg) *						
		W ^1^	C ^2^	FA ^3^	NS ^4^	RS ^5^	SF ^6^	SP ^7^
OPC	0.40	323	807	-	1090	-	-	0.16
OPCF		323	807	-	1090	-	20.12	0.81
F25S50F		323	605	202	545	545	20.12	0.81

^1^ Water, ^2^ Cement, ^3^ Fly ash, ^4^ Natural sand, ^5^ Recycled sand, ^6^ Steel fiber, ^7^ Superplasticizer. * Air content for calculating the mix proportion was assumed to be 2.0%.

**Table 3:** Chemical and physical characteristics of cementitious materials.

Parameter	Type I Portland Cement	Class F Fly Ash
SiO_2_ (%)	33.44	50.72
Al_2_O_3_ (%)	15.03	20.73
Fe_2_O_3_ (%)	0.57	6.37
CaO (%)	44.12	3.61
Free CaO (%)	0.82	-
MgO (%)	3.55	1.08
SO_3_ (%)	3.45	0.54
Ig. loss (%)	1.27	3.04
C_3_S (%)	61.24	-
C_2_S (%)	12.09	-
C_3_A (%)	13.14	-
C_4_AF (%)	6.12	-
Blaine (cm^2^/g)	3500	3990

**Table 4:** Physical properties of fine aggregates.

Type	Density (g/cm^3^)	Water Absorption (%)	Fineness Modulus
Natural sand	2.59	0.76	2.44
Recycled sand	2.44	4.32	2.99

**Table 5:** Physical properties of micro steel fiber.

Dia. (mm)	Length (mm)	Elastic Modulus (GPa)	Tensile Strength (MPa)
0.18–0.23	12–14	206	2580

**Table 6:** Twenty-eight-day strength characteristics of cement composites.

Mix	Compression			Bending Strength (MPa)	Tensile Strength (MPa)
	Strength (MPa)	Elastic Modulus (GPa)	Poisson’s Ratio		
OPC	51.9	24.12	0.16	5.54	2.23
OPCF	61.4	22.49	0.16	6.39	2.62
F25S50F	53.9	16.09	0.19	6.43	2.62

**Table 7:** Results of flexural tests.

Specimen	Peak Load (kN)			Deflection at Peak Load (mm)	Denting Depth at Peak Load (mm)	Dissipated Energy (kN·m)		
	Test (1)	AISC (2)	(1)/(2)			by Deflection	by Denting	Total
STC	202.42	201.20	1.01	71.10	4.05	15.939	0.872	16.811
STF	207.33	201.08	1.03	82.50	6.73	17.989	1.145	19.134
STFR	203.98	201.18	1.01	79.16	9.38	18.416	1.656	20.072

## Data Availability

Data are available upon request.
